# Radiology's Ionising Radiation Paradox: Weighing the Indispensable Against the Detrimental in Medical Imaging

**DOI:** 10.7759/cureus.41623

**Published:** 2023-07-10

**Authors:** Reabal Najjar

**Affiliations:** 1 Medical Imaging, Canberra Hospital, Australian Capital Territory (ACT) Health, Canberra, AUS

**Keywords:** diagnostic necessity, alara principle, dose optimisation, patient safety, radiation protection, radiology, medical imaging, ionising radiation

## Abstract

Ionising radiation stands as an indispensable protagonist in the narrative of medical imaging, underpinning diagnostic evaluations and therapeutic interventions across an array of medical conditions. However, this protagonist poses a paradox - its inestimable service to medicine coexists with an undercurrent of potential health risks, primarily DNA damage and subsequent oncogenesis. The narrative of this comprehensive review unfurls around this intricate enigma, delicately balancing the indispensable diagnostic utility against the non-negotiable commitment to patient safety.

In this critical discourse, the intricacies of ionising radiation are dissected, illuminating not only its sources but also the associated biological and health hazards. The exploration delves into the labyrinth of strategies currently deployed to minimise exposure and safeguard patients. By casting light on the scientific nuances of X-rays, computed tomography (CT), and nuclear medicine, it traverses the complex terrain of radiation use in radiology, to promote safer medical imaging practices, and to facilitate an ongoing dialogue about diagnostic necessity and risk.

Through a rigorous examination, the pivotal relationship between radiation dose and dose response is elucidated, unravelling the mechanisms of radiation injury and distinguishing between deterministic and stochastic effects. Moreover, protection strategies are illuminated, demystifying concepts such as justification, optimisation, the As Low As Reasonably Achievable (ALARA) principle, dose and diagnostic reference levels, along with administrative and regulatory approaches.

With an eye on the horizon, promising avenues of future research are discussed. These encompass low-radiation imaging techniques, long-term risk assessment in large patient cohorts, and the transformative potential of artificial intelligence in dose optimisation. This exploration of the nuanced complexities of radiation use in radiology aims to foster a collaborative impetus towards safer medical imaging practices. It underscores the need for an ongoing dialogue around diagnostic necessity and risk, thereby advocating for a continual reassessment in the narrative of medical imaging.

## Introduction and background

Medical imaging in modern healthcare

Medical imaging constitutes an indispensable cornerstone of contemporary healthcare, transfiguring the trajectory of disease diagnosis and management. Its pertinence transcends mere visualisation of anatomic structures, extending to early disease detection, definitive diagnosis, informed treatment planning, and efficacy monitoring, and reducing the reliance on invasive procedures [1]. Grounded in these capabilities, imaging modalities such as radiography and computed tomography (CT) provide clinicians with pivotal insights into patient health, enabling timely, targeted, and effective therapeutic interventions. Magnetic resonance imaging (MRI), a radiation-free technique, further complements these modalities, particularly when radiation exposure is a concern.

The ubiquitous integration of medical imaging in healthcare systems has been attributed to its potential to improve patient outcomes, optimise resource utilisation, and minimise overall healthcare costs [2]. Technological advancements in image processing, telemedicine, and artificial intelligence have further augmented its clinical utility and accessibility, particularly in remote and resource-constrained settings [1]. Despite such compelling benefits, this technology-driven paradigm shift raises significant concerns regarding patient safety, particularly in the realm of radiation-induced adverse effects [3]. Consequently, striking an optimal balance between diagnostic necessity and radiation safety remains an enduring challenge.

The role of ionising radiation in radiology

Ionising radiation serves as the foundation of several major imaging modalities within radiology, which are indispensable in contemporary medicine for enabling the non-invasive visualisation of internal structures and processes. Specifically, X-ray-based technologies such as radiography, mammography, CT, and fluoroscopy, which account for around 85% of all imaging examinations, leverage the differential absorption properties of X-rays to generate detailed anatomical images [4]. Conversely, nuclear medicine techniques, such as positron emission tomography (PET) and single-photon emission computed tomography (SPECT), contributing to approximately 15% of imaging studies, utilise ionising gamma rays emitted from radioisotopes to offer invaluable metabolic and functional information [5].

Such techniques are vital in the early detection, accurate diagnosis, and successful treatment of various diseases, underpinning the crucial role of radiology in advancing patient care. Despite their remarkable diagnostic capabilities, these imaging techniques expose patients to ionising radiation, potentially increasing cancer incidence [6]. Notwithstanding the intrinsic risks, the judicious use of ionising radiation in radiology remains integral to modern healthcare owing to its unique diagnostic capabilities.

Biological impact of low-dose radiation

Despite the indisputable diagnostic efficacy of radiological imaging, exposure to ionising radiation presents potential risks to patient safety. As such, it is incumbent to shed light on the possible hazards patients may encounter. Ionising radiation, by its very nature, possesses the capacity to inflict harm at the cellular level, exhibiting both immediate and long-term risks [7]. Acutely, high-dose exposure may induce radiation sickness, manifesting as nausea, vomiting, and, in severe cases, organ failure and death. Nevertheless, such extreme scenarios are practically non-existent in the realm of diagnostic radiology.

The greater concern pertains to long-term risks - principally, the increased likelihood of malignancies and germline mutations that may propagate the risk to future generations following prolonged or recurrent low-dose exposure [8]. Although empirical evidence substantiates this association, quantifying the precise risk remains a complex endeavour due to latency periods and the multifactorial causality of cancers [7]. Moreover, individual radiosensitivity - influenced by genetics, age, and sex - further complicates risk assessment. The challenge, therefore, necessitates a thorough equipoise between the diagnostic indispensability of radiology and the potential adverse health outcomes, accentuating the significance of ongoing risk-benefit analyses in radiological practice.

## Review

Understanding ionising radiation: a brief overview

Ionising radiation, a crucial component in modern diagnostic and therapeutic modalities, possesses the capacity to ionise atoms by displacing electrons from their orbits [9]. In the context of medical imaging, the types of radiation that are most relevant include gamma (γ) rays, X-rays, and, in certain instances, neutron radiation, each with unique physical characteristics and interactions that dictate their utility and biological impact [10].

**Figure 1:**
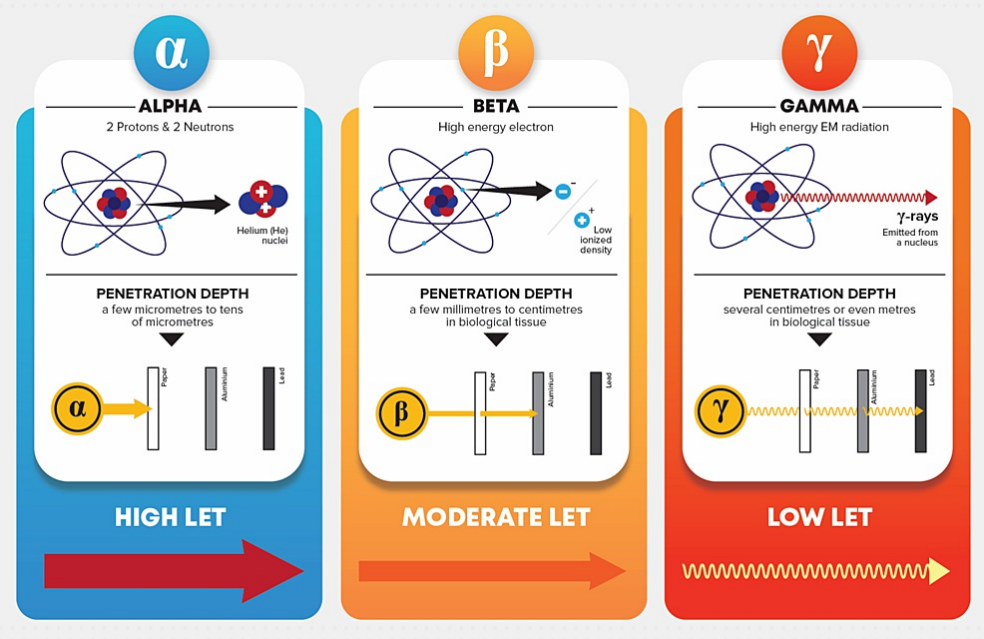
Alpha (α), beta (β), and gamma (γ) radiation and their unique characteristics. Image credit: Reabal Najjar

Gamma rays and X-rays, categorised as high-energy electromagnetic waves, lack mass or charge, endowing them with the potential for deep penetration. This ability results in sparse ionisations along their path, rendering them ideal for the needs of medical imaging and radiotherapy (Figure 1) [10].

Though alpha (α) and beta (β) particles, which emanate primarily from natural radioactive decay, are not typically utilised in diagnostic radiology, however, they are of particular note in nuclear medicine and radiation therapy as they serve to offer a broader context to the diverse range of ionising radiations [10]. Alpha particles, essentially helium nuclei bearing a +2 charge, exhibit low penetration and high linear energy transfer (LET), causing substantial ionisations within a small volume [10]. Their damage potential is particularly notable when emitted intracellularly or proximal to delicate structures primarily due to their significant mass and charge [11]. Beta particles, electrons with a -1 charge, are characterised by their reduced mass and charge, which afford them more substantial penetration capabilities within matter and moderate LET [10].

Radiation measurements employ the Gray (Gy) for absorbed dose, Sievert (Sv) for biologically effective dose, and Becquerel (Bq) for radioactivity [12]. The Sievert is the standard unit used to measure the biological effect of radiation on the human body. The millisievert (mSv) is equal to one-thousandth of a Sv and is typically used to denote smaller doses, such as those encountered in medical imaging or background radiation [12].

The International Commission on Radiological Protection (ICRP) recommends dose limits of 1 mSv per year, averaged over five years, for public exposure. On the other hand, for occupational exposure, the recommendation is 20 mSv annually, 20 mSv per year, also averaged over a five-year period, but with no single year exceeding 50 mSv [13].

The annual effective doses stemming from natural background radiation vary geographically, with a world-averaged annual exposure dose of 2.4 mSv. However, regional disparities are significant: Kuwait records as low as 1.05 mSv, the Netherlands 1.5 mSv, and Cyprus 1.6 mSv. Conversely, the figures reach as high as 7 mSv in Brazil, 6.4 mSv in China, and 6 mSv in Iran [13]. In Australia, the average annual dose approximates 1.5 mSv, whereas the annual doses for the United States and Canada are typically around 3.1 mSv and 1.8 mSv, respectively [13].

**Figure 2:**
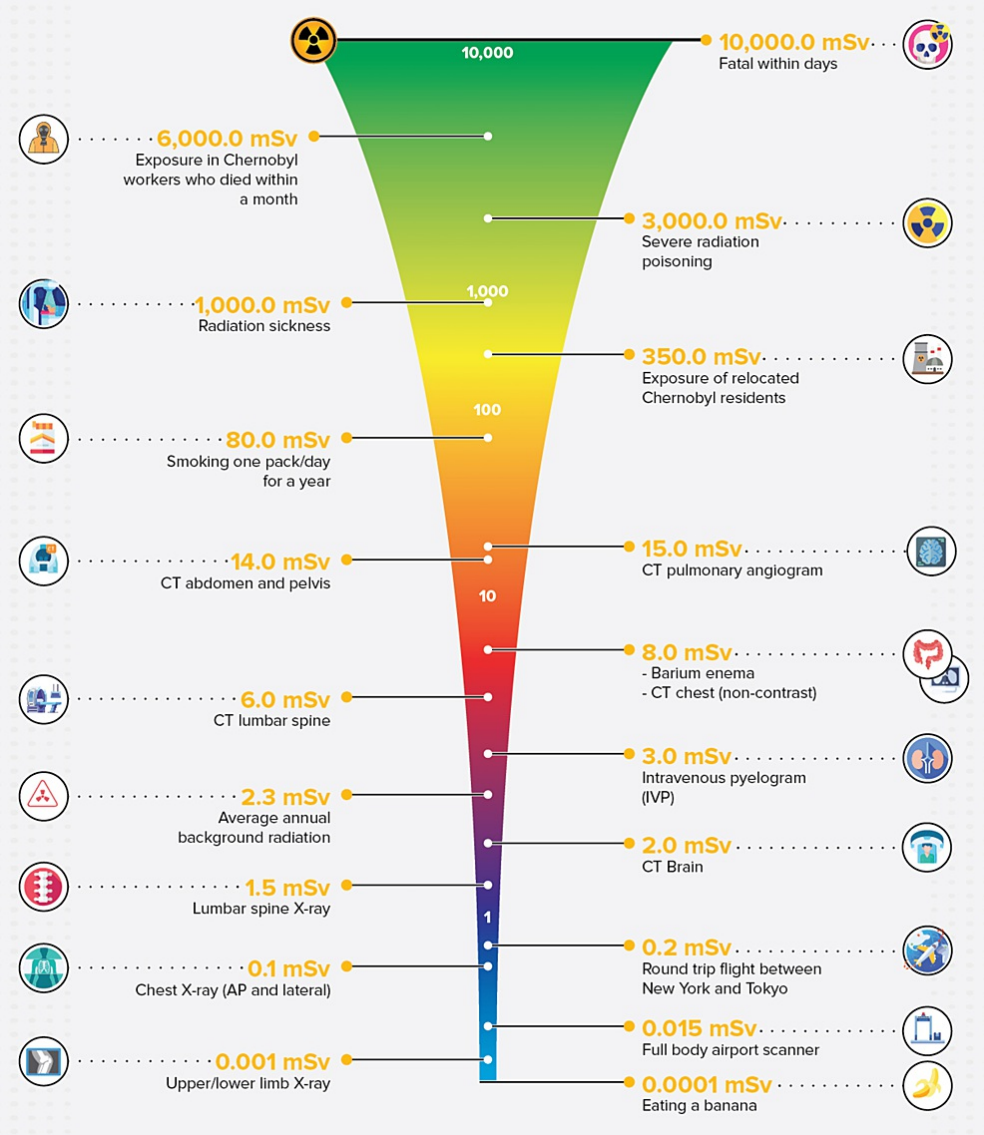
Comparative overview of different sources of ionising radiation. Image credit: Reabal Najjar

In addition to natural sources, medical exposures contribute an additional 1.7 mSv on average to the annual dose in Australia [14]. Most professionals working in radiation facilities do not typically experience no occupational exposure. However, among those who do, the average annual dose aligns with the public limit of 1 mSv. Certain specialised fields, such as nuclear medicine and cardiology, may encounter higher occupational doses, with annual averages of around 3 mSv (Figure 2) [15]. The differences in these figures across regions highlight the necessity of localised data in assessing radiation exposure and potential health risks.

Ionising radiation sources can be classified into natural and artificial categories. Natural radiation, commonly referred to as background radiation, primarily derives from cosmic radiation, terrestrial radon, and internal human radionuclides [16]. Artificial sources, on the other hand, encompass human-made contributions from nuclear power generation, industrial applications, and importantly, medical procedures, the latter increasingly contributing to per capita radiation dose [17]. Therefore, understanding ionising radiation forms the foundation for exploring its implications in healthcare.

Ionising radiation in diagnostic and interventional imaging

X-rays

Radiography forms the bedrock of diagnostic imaging, capitalising on the penetrative capabilities of X-rays on human tissues and organs to generate two-dimensional planar images of varying contrasts. The amount of radiation used can differ markedly from less <0.01 mSv for limb radiographs, 0.1 mSv for a chest radiograph, to 0.4 mSv for a mammogram breast X-ray [3]. This variation stems from the nature of the specific examination and patient characteristics, which may impact image quality and patient safety. The critical role of X-rays is underscored by their usage in over 60% of all thoracic evaluations and approximately 80% of mammographic examinations conducted worldwide [3,7].

Fluoroscopy is a derivative of X-ray technology that provides real-time visualisation crucial to several clinical procedures. This includes those in interventional radiology and vascular surgery, where it aids in procedures such as angioplasty, stent placement, and embolisation. Given its dynamic nature, fluoroscopic procedures can involve doses ranging from less than 1 mSv for a simple study to potentially over 80 mSv for complex or prolonged interventional procedures [3].

One notable application of fluoroscopy is gastrointestinal studies like barium enema examinations, which typically administer a radiation dose ranging from 3 to 8 mSv. However, complex interventional procedures such as coronary angioplasty, peripheral artery angioplasty, and stenting for peripheral artery disease can involve significantly higher doses, around 15-20 mSv. Transjugular intrahepatic portosystemic shunt (TIPS) procedures can reach almost 75 mSv, contingent on the procedure's complexity and duration [3,10].

In the field of vascular surgery, catheter-directed thrombolysis for deep vein thrombosis or pulmonary embolism can involve radiation doses of around 20 to 40 mSv [3,18]. Endovascular aneurysm repair (EVAR) procedures used for the treatment of abdominal aortic aneurysms typically involve radiation doses ranging from 20 to 70 mSv depending on the complexity of the case. A more complex procedure, such as fenestrated endovascular aneurysm repair (FEVAR) for complex aortic aneurysms, may even exceed 80 mSv due to the need for precise navigation and deployment of fenestrated grafts [3,18].

Computed Tomography (CT)

CT scans transcend standard imaging, producing intricate cross-sectional images using a rotating X-ray beam coupled with detector arrays [1]. Despite its diagnostic prowess, the increased complexity and sophistication of CT imaging also introduce higher radiation doses, delivering a radiation dose generally between 2 and 20 mSv [1,6].

The total radiation dose administered in a CT procedure is a function of multiple variables, including scan parameters like tube current and voltage, patient size, and the extent of the anatomical region scanned [10]. A typical head CT scan might deliver an effective dose of about 2 mSv, while a CT scan of the abdomen and pelvis may administer a dose closer to 14 mSv. A whole-body CT scan can involve a higher dose, around 20 mSv [6].

The dual-energy CT, a significant improvement over its traditional counterpart, utilises two distinct X-ray energy levels, distinguishing tissues based on their energy-dependent attenuation characteristics and enhancing diagnostic precision. In terms of dose, dual-energy CT scans typically provide radiation doses comparable to or slightly higher than those of single-energy CT scans, but the added information they provide can potentially reduce the need for additional imaging studies, offsetting the slightly higher doses they may impart and minimising the cumulative radiation dose to the patient [18].

Nuclear Medicine

Nuclear medicine, another key player in diagnostic imaging, provides functional insights into the body. Radiation dose in nuclear medicine procedures is unique as the radiotracer administered to the patient determines the dose [19]. Positron Emission Tomography (PET) scans utilise a radiotracer, emitting positrons upon decay, resulting in the generation of gamma photons via the collision of electrons, which are subsequently captured and reconstructed into images reflecting metabolic or physiological activity [19].

The typical radiation dose ranges from 5 to 20 mSv, depending on the specific radiotracer used and the patient's body size. For a standard FDG-PET scan, typically used in oncology, the radiation dose is around 7 mSv. Single Photon Emission Computed Tomography (SPECT), which employs radiotracers that emit gamma radiation directly, is notably useful for assessing cardiac function and mapping cerebral activity in neurological conditions. SPECT myocardial perfusion imaging can administer an average radiation dose of around 12 mSv [19].

Ionising radiation in imaging-based therapeutics

External Beam Radiotherapy

Ionising radiation, with its profound influence on biological structures, is an instrumental component of various therapeutic modalities. Its capacity to induce DNA damage and instigate apoptosis, or programmed cell death, has been well-established [20].

External beam radiotherapy (EBRT), which employs high-energy rays directed at tumours from an extracorporeal source, involves the delivery of a high radiation dose, often in the range of 20 to 70 Gy (20,000 to 70,000 mSv), directly to the tumour site. For instance, a typical course of EBRT for lung cancer could involve total doses of around 60 Gy (60,000 mSv), delivered in multiple fractions. Therefore, precise calculation and delivery of this dose are fundamental to achieving therapeutic success and limiting side effects [21].

Recent advancements in EBRT include the introduction of three-dimensional conformal radiation therapy (3D-CRT). This innovative technique leverages sophisticated imaging technology and computer-assisted treatment planning to contour the radiation beams precisely to the tumour's spatial profile. This approach enhances the therapeutic ratio by facilitating higher radiation dose administration directly to the tumour while maintaining the integrity of adjacent healthy tissue [22].

Brachytherapy

Stepping beyond the bounds of external methods, brachytherapy employs ionising radiation internally by the direct insertion of radioactive sources into the tumour site. The radiation dose in brachytherapy is highly localised, thus limiting exposure to surrounding healthy tissues [23]. Techniques differ based on the rate at which the dose is delivered: low-dose rate (LDR) brachytherapy gradually emits radiation over a period of days or weeks, while high-dose-rate (HDR) brachytherapy delivers powerful radiation within a span of minutes to hours [23].

The radiation dose can range from a few Gy to several hundred Gy, depending on the technique and clinical scenario, utilising either temporary or permanent implants. For instance, LDR brachytherapy for prostate cancer might deliver around 145 Gy (145,000 mSv) to the prostate over several days [24].

Radiopharmaceuticals

Radiopharmaceuticals are revolutionising the field of medical therapeutics by marking an increasing significance in the application of ionising radiation. Comprising radioactive isotopes conjugated to biologically active molecules, these compounds are devised to selectively target pathological sites by exploiting unique physiological or metabolic characteristics inherent to the site of disease [25].

In general, radiopharmaceuticals deliver a radioisotope to a specific site in the body by linking the isotope to a molecule with a high affinity for that site. For instance, iodine-131 (I-131) is systemically used for thyroid cancers, as thyroid cells naturally uptake iodine. I-131 is taken up and decays within the thyroid cells, irradiating them from the inside and thus selectively targeting cancer [25].

The types of radiopharmaceuticals vary from I-131 for thyroid cancers to yttrium-90 (Y-90) microspheres for liver malignancies. Other examples include phosphorus-32 (P-32) for polycythemia vera and chronic myelogenous leukemia, and strontium-89 (Sr-89) and samarium-153 (Sm-153) for palliation of bone pain in cases of metastatic bone disease [25].

The radiation dose delivered via radiopharmaceuticals can vary greatly, from a few mSv to several tens of Gy (several thousand mSv), depending on the isotope used and the clinical application. As a case in point, the systemic delivery of I-131 for thyroid cancers can administer a dose of approximately 200 Gy (200,000 mSv) to the thyroid tissue, while the effective dose to the whole body is significantly lower, approximately 2 mSv [25].

Health implications of ionising radiation: dosimetry and biological impacts

Radiation Dose and Dose-Response Relationship

Radiation dose forms an integral part of radiobiological studies and risk assessments. It refers to the quantity of ionising radiation absorbed by an object or person, which can be primarily described in three ways: radiation dose, absorbed dose, and effective dose [10].

The radiation dose, frequently referred to as exposure, describes the amount of ionising radiation present in the environment, measured in Roentgen (R) [10]. Conversely, the absorbed dose, delineated in Gy, accounts for the energy deposited by ionising radiation per unit mass of matter, commonly biological tissue [10]. Notably, the effective dose, represented in Sv, provides the most comprehensive depiction of the biological harm resultant from radiation exposure [10]. It factors in the absorbed dose, the radiation type, and the radiosensitivity of the exposed tissues or organs, providing a more nuanced estimate of stochastic health risks across various radiation types and exposure scenarios.

To ascertain the implications of varying radiation doses, dose-response relationships are employed, with a particular emphasis on the prevalent Linear No-Threshold (LNT) model [26]. This model operates under the assumption that any level of radiation exposure carries some degree of risk, suggesting a linear correlation between radiation dose and the probability of an effect, without any safe level of exposure. Expanding upon this, the LNT model's simplicity and conservativeness have largely driven its prevalence in radiobiological studies and regulatory measures. However, it is worth noting that this model is not without contention. Some argue that it may overestimate the risk at low doses, prompting ongoing scientific debate [26].

Contrarily, other dose-response models, including the threshold and hormesis models, propose alternative relationships. The threshold model posits the existence of a dose below which no detrimental effects are expected, implying a dose 'safety zone', offering a stark contrast to the LNT model [27]. The hormesis model, on the other hand, suggests that low levels of radiation might confer a protective effect. This concept, known as radiation hormesis, proposes that low-dose radiation can stimulate cellular repair mechanisms, potentially enhancing biological function and decreasing the risk of certain diseases [27]. Despite these proposed benefits, the hormesis model remains controversial, and it is not widely accepted in the scientific community or incorporated into radiation protection guidelines.

Finally, the concept of cumulative dose, which is a summative measure of total radiation exposure over time, holds significance in scenarios of chronic exposure. This measure highlights the potential risks of repeated or continuous low-dose exposures that may collectively engender significant long-term health impacts [27].

Radiation-Induced Genotoxic Damage

Ionising radiation is a powerful genotoxic agent, damaging DNA both directly, through atomic disruption within DNA molecules and indirectly, through radiolysis of cellular water molecules [28]. The latter generates reactive free radicals, such as hydroxyl radicals, that instigate oxidative base damage, strand breaks, and DNA cross-links. Concurrently, direct damage results from molecular ionisation and excitation, courtesy of the ionising nature of the radiation, leading to DNA strand severance [29].

These forms of damage notably encompass single and double-strand breaks, base modifications, and DNA-protein crosslinks, with double-strand breaks (DSBs) representing the most severe injury due to their potential to induce chromosomal aberrations and genomic instability [29]. Any inaccuracies in DSB repair can significantly amplify the risk of malignant transformation.

Cellular responses to radiation damage pivot on a delicate equilibrium of defence mechanisms, including DNA repair, cell cycle arrest, and programmed cell death. This balanced protective suite shapes the cell's fate and influences the long-term risk of radiation-induced pathologies [30]. While robust repair mechanisms typically mitigate low-dose radiation's oncogenic potential, inaccuracies or persisting aberrations can instigate mutation accumulation and chromosomal disruptions, thus fostering cellular transformation and malignant proliferation [31].

These intricacies underscore the nuanced interplay of radiation-induced damage, DNA repair, and cellular responses, providing critical insights into radiation-associated risks and potential mitigation strategies.

Stochastic Effects: Oncogenesis and Heritable Mutations

Stochastic effects, characterised by their probabilistic occurrence and non-threshold nature, encapsulate two principal sequelae of low-dose radiation exposure: induction of cancer and heritable effects [32]. Ionising radiation can provoke DNA mutations, triggering carcinogenesis by disrupting cell regulatory genes. Heightened lifetime risks of various cancers predominantly reflect these stochastic effects. Yet, their manifestation is non-deterministic and subject to a suite of individual factors such as age, sex, genetic predisposition, and specifics of radiation exposure [32].

Collaborative research by the European Society of Radiology (ESR) and the European Federation of Radiographer Societies (EFRS) highlights the necessity of acknowledging both linear and non-linear dose-response relationships in assessing radiation-induced malignancies and the role of individual susceptibility factors [33]. The most salient evidence of these effects derives from historical epidemiological studies. Notably, atomic bomb survivors and populations exposed to substantial radiation doses offer compelling evidence of a distinct dose-response relationship [34,35]. However, the actual manifestation hinges on an intricate interplay of individual factors and external conditions, underscoring the multifaceted dynamics and complexities of stochastic effects.

The biological interaction with radiation unleashes a cascade of consequential biophysical and biochemical events, such as the production of reactive oxygen species and free radicals, which jeopardise cellular integrity [29]. This response, however, is not universal, contingent upon numerous variables, including the nature and intensity of radiation, exposure rate, and the individual's unique genetic and physiological makeup [7]. A corollary concern lies in potential heritable changes, given that germ cell mutations can propagate across generations, potentially inciting genetic disorders [36]. Although less prevalent, heritable effects persist as a significant consideration in radiation protection, mandating nuanced understanding and ongoing research.

The "bystander effect" adds a layer of complexity to this narrative, suggesting that non-irradiated cells can emulate the responses of their irradiated counterparts [37]. This emphasises the critical role of intercellular communication in shaping the biological milieu following radiation exposure. Importantly, this insight carries profound implications for understanding and mitigating the broader impact of ionising radiation within tissue and organ systems.

Deterministic Effects: Tissue and Organ Reactions

Radiation-induced deterministic effects, also known as tissue reactions, occur in a threshold-dependent, dose-proportional manner, markedly differing from stochastic counterparts. The manifestation and severity of these effects are contingent upon exceeding a radiation dose threshold [38].

Various acute deterministic effects impact tissues and organs with high cellular turnover rates, such as the skin, bone marrow, and gastrointestinal tract. Their radiosensitivity triggers symptoms within hours of exposure, including erythema, desquamation, gastrointestinal discomfort, and significant depletion of the haematopoietic system, resulting in lymphopenia and increased infection risk [38]. Collectively, these symptoms constitute Acute Radiation Syndrome (ARS), the severity of which is determined by the degree and extent of exposure and is typically apparent within hours to days post-exposure [39]. Despite being primarily an occupational risk and rare in diagnostic radiology due to safety regulations, ARS underscores the inherent vulnerabilities of cellular and tissue structures to ionising radiation, exemplifying the nature of deterministic effects.

Radiosensitive tissues exhibit varied responses to radiation exposure; bone marrow may develop haematopoietic syndrome at doses above 0.5 Gy, the gastrointestinal tract may exhibit syndrome at doses exceeding 30 Gy, while the central nervous system might develop cerebrovascular syndrome at doses surpassing 50 Gy [40].

**Figure 3:**
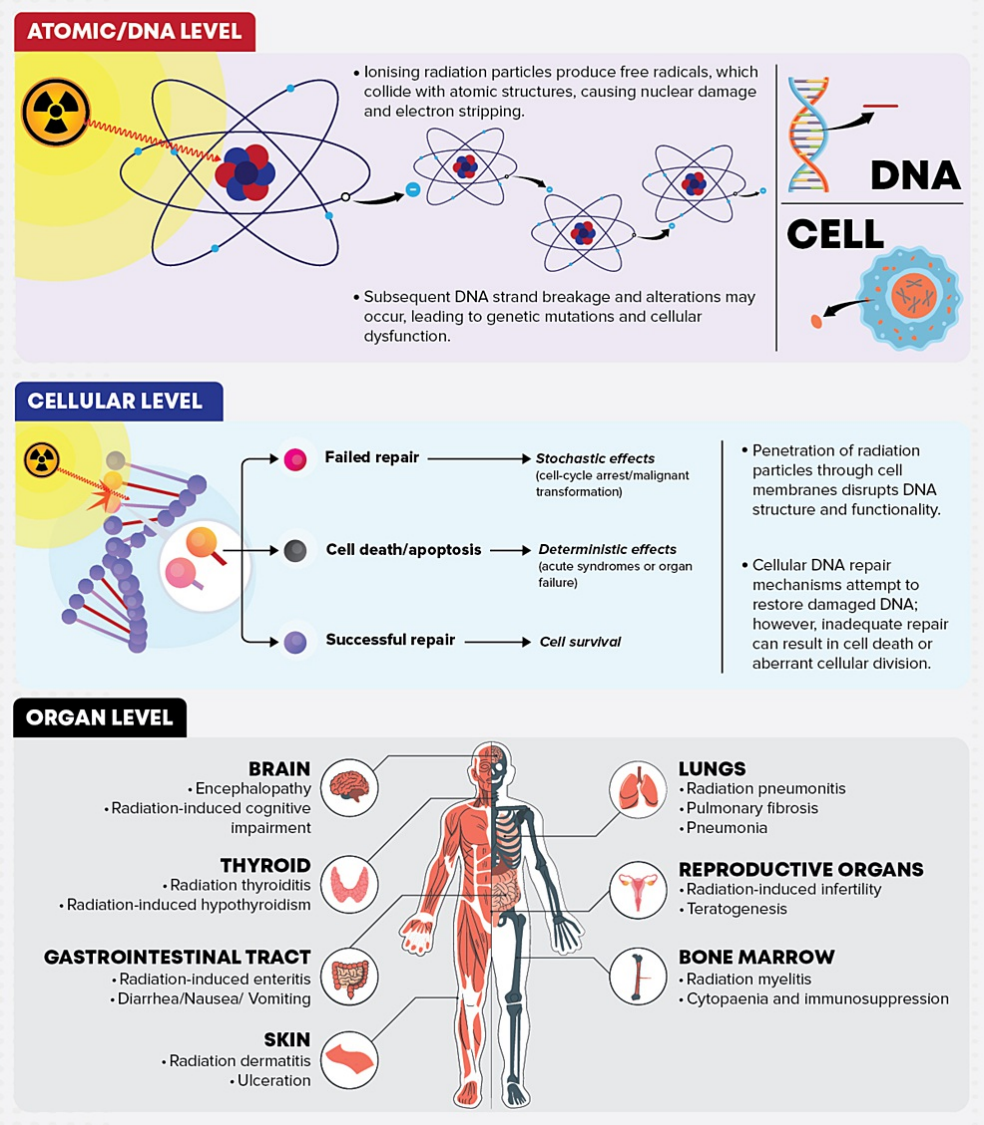
The biological impact of ionising radiation at an atomic/DNA, cellular, and organ level. Image credit: Reabal Najjar

Tissue reactions, whether early or late onset, have profound implications for a patient's quality of life and frequently require medical intervention. These effects, while absent at lower doses, indicate significant morbidity at high doses, underlining the necessity for stringent control measures to minimise radiation exposure and safeguard patient health (Figure 3) [40].

Individual Risk Factors and Vulnerable Populations

The fundamental necessity to optimise radiation safety in medical imaging is deeply rooted in understanding the complex nexus of individual risk factors and the augmented susceptibility of particular cohorts to ionising radiation. These cohorts span from paediatric patients, whose heightened radiosensitivity and extended life expectancy significantly increase their latent malignancy risk, to women who are especially prone to breast and thyroid cancers, thus necessitating vigilant thoracic and CT imaging utilisation [39,41-43].

Pregnant women warrant extra attention due to the acute radiation sensitivity of the developing foetus, potentially leading to adverse health outcomes [44,45]. Among other high-risk groups are immunocompromised individuals and those genetically predisposed to radiation sensitivity, such as carriers of Down's Syndrome or Ataxia Telangiectasia [46,47]. Such individuals may necessitate not only diligent dose management and monitoring but also an exploration of alternative diagnostic methodologies.

Our understanding of vulnerability to ionising radiation also extends beyond biological factors to social determinants. Socioeconomic status, educational levels, and access to quality healthcare may magnify the risk for certain populations, exacerbating radiological health disparities [48]. Therefore, an equitable approach encompassing personalised medicine, tailored imaging protocols, and patient-centred care is of utmost importance.

Comprehending the interplay of factors like age, sex, genetic predisposition, and previous radiation exposure is instrumental in enhancing radiation safety, fostering nuanced and optimising patient care.

Principles of radiation protection

Justification

Justification, a cardinal principle in radiation safety in radiology, posits that any exposure to ionising radiation must confer a net overall benefit [13]. Essentially, the diagnostic or therapeutic gains must significantly outweigh potential detrimental effects. Given the paradox of radiation - a double-edged sword beneficial for diagnosis and therapy, yet potentially harmful - the adoption of this principle becomes imperative for patient safety [49].

In the healthcare context, the justification principle assumes two tiers. At the first level, a particular radiological procedure is deemed beneficial for managing a broad category of clinical conditions. Regulatory bodies, such as the Australian Radiation Protection and Nuclear Safety Agency (ARPANSA), use scientific evidence to form these general justifications [15].

The second level of justification comes into play at the point of care. Here, the healthcare professional must meticulously ascertain that the procedure is indeed advantageous for the individual patient. Personalised aspects, including the patient's clinical history, current health status, and specific diagnostic or therapeutic needs, are meticulously considered. Moreover, alternatives that expose the patient to less or no ionising radiation are contemplated [50].

This process of rigorous justification requires continuous, collaborative efforts from radiologists, referring physicians, and medical physicists. They work synergistically to ensure the patient's well-being, underlining that the benefits procured surpass the potential risk. Consequently, the principle of justification serves not only as an ethical obligation but also as a cornerstone for maintaining a high standard of patient-centred care in radiology [51].

Protocol Optimisation

Radiological protection is deeply rooted in the ALARA (As Low As Reasonably Achievable) philosophy, which delicately balances diagnostic efficiency with minimal radiation exposure, given prevailing societal and economic conditions [52]. This essence of optimisation, entailing protocol selection and technique adjustment, forms the cornerstone of this principle.

Protocol selection, a crucial aspect of this strategy, involves meticulously selecting an imaging procedure tailored to each specific clinical scenario. While ultrasound or MRI can entirely circumvent ionising radiation in certain contexts, there are situations where radiological imaging remains invaluable [50]. In such cases, an astute choice between imaging modalities and protocols, such as between single-phase and multiphase CT, can lead to significant radiation reduction.

Adjusting technical parameters represents a secondary, yet pivotal, facet of optimisation. The intricate balance between radiation dose and image quality calls for the nuanced manipulation of parameters like tube current, voltage, and exposure time [53]. This complexity demands a thorough understanding of the imaging equipment in conjunction with the individual patient's characteristics.

**Figure 4:**
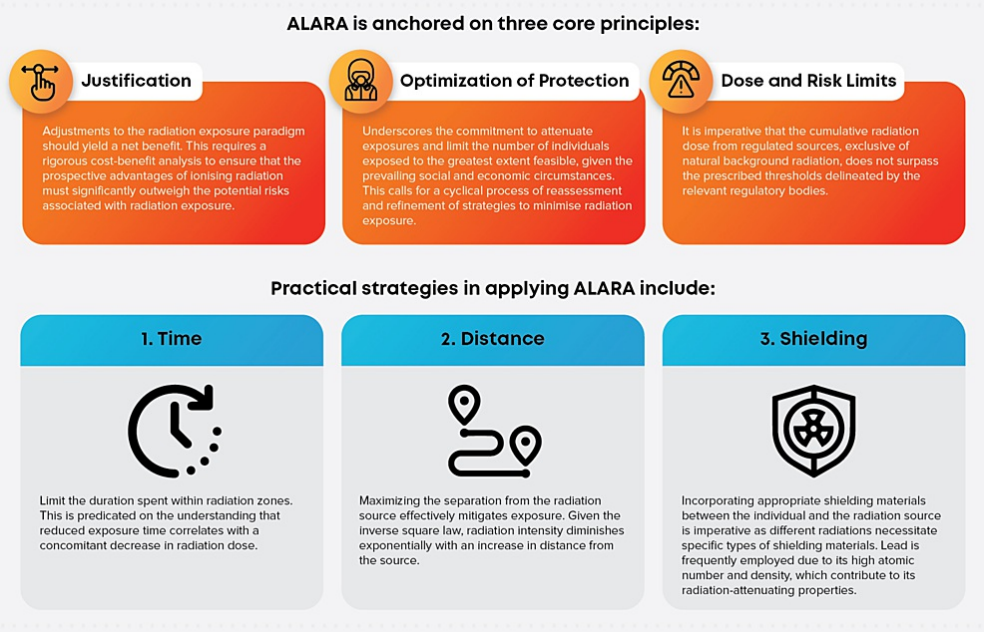
The ALARA (As Low As Reasonably Achievable) principle in radiation protection. Image credit: Reabal Najjar

Intrinsically, the ALARA philosophy pivots around three cardinal tenets. Time, in which limiting the duration within radiation zones, correlates with reduced radiation dose. Distance, where maximising the space from the radiation source, significantly mitigates exposure, highlighting the relevance of the inverse square law. Shielding, which requires appropriate materials, often lead, is placed between the individual and the radiation source, varying with the radiation type due to distinct attenuation properties (Figure 4) [52].

Enhancing the optimisation process further involves the adoption of dose-reduction technologies such as automatic exposure control through collimation, which involves adjusting the tube current-time product (mAs) and tube voltage (kVp) according to patient size and diagnostic requirements [54,55]. Additionally, iterative reconstruction techniques can be utilised, which employ statistical models in conjunction with complex computing algorithms to reconstruct radiological images from scan data.

Unlike traditional filtered back projection methods, which require higher radiation doses for quality imaging, iterative techniques enable reduced dose acquisitions [56]. These methods work by an initial assumption of the object being imaged, which is then projected and compared to the measured projection data. The initial assumption is then iteratively refined based on the deviation from the actual measurement until the discrepancy is minimised. The result is a reconstructed image that, despite being based on lower radiation dose data, does not compromise diagnostic quality [56].

Paired with robust quality assurance programs, these technologies offer a robust defence against unnecessary radiation exposure, underlining the radiological community's commitment to patient welfare and steadfast adherence to the ALARA principle.

Diagnostic Reference Levels

Radiation protection requires meticulous management of radiation dosage, ensuring exposure under typical conditions does not exceed specified thresholds. These thresholds, referred to as Diagnostic Reference Levels (DRLs), underpin radiological protection practices, essential for safeguarding patients from excessive ionising radiation [13].

The effective dose is a crucial metric in this process, constructed via computational models to capture tissue-specific radiosensitivity. Designed to assess the potential biological impact and consequent risk, this measure is vital for modulating radiation exposure [57]. Alongside dose reference levels, DRLs play a significant role in controlling radiation dosage in medical imaging environments as they support diligent surveillance and promote adherence to the ALARA principle, simultaneously ensuring diagnostic efficacy [58].

DRLs, established by various international and national bodies, represent recommended upper dose limits for a majority of examinations for specific imaging procedures [13-15,59]. They are derived from broad-based dose distribution analyses across diverse institutions, indicative of consensus on optimal practices. DRLs are typically set at the 75th percentile of the dose distribution from a survey of practices [57,58]. This means that if a facility's standard radiation dose for a particular procedure consistently exceeds the DRL, it signals the need for review and potential optimisation of protocols.

Dose reference levels serve as quality assurance tools within individual facilities, pinpointing inefficient practices that may be improved for greater efficiency and patient safety. Regular dose audits, comparison with established standards, and necessary adjustments constitute the successful implementation of DRLs and dose reference levels. Such a process is imperative for performance benchmarking and validation of compliance with ALARA principles. Furthermore, these tools can be employed for patient-specific dose calculations, thereby aiding personalised risk assessments and facilitating informed decision-making [57].

In emergency or controllable exposure situations, reference levels indicate the dose or risk level exceeding which exposures are considered unacceptable and under which protection optimisation is mandated [58]. Dose constraints function as a prospective restriction on individual doses from a specific source, acting as the upper limit on dose optimisation. Concurrently, dose limits specify the maximum effective or equivalent dose permissible from planned exposure situations [58]. Collectively, these guidelines oversee radiation use, maintaining a risk-benefit equilibrium and reinforcing the primacy of patient safety in radiology.

Administrative and Regulatory Approaches

Ensuring the safe use of ionising radiation in radiology requires adherence to international and national regulatory guidelines, along with robust facility policies and procedures. The ICRP, IAEA, European Commission, ARPANSA, and World Health Organization (WHO) collectively furnish global and regional standards for the prudent application of medical imaging technologies. These guidelines establish critical boundaries for radiation exposure, contributing to the formulation of local radiation safety protocols and goals [12-15,59].

Akin to orchestrating a symphony, managing radiation safety within healthcare facilities necessitates harmonious collaboration, with the radiation safety officer (RSO) wielding the conductor's baton. This vital role encompasses orchestrating administrative procedures, engaging in regular intercommunication with the radiation safety committee, and monitoring adherence to prescribed safety measures [60]. The RSO carries the responsibility for the overall coordination, implementation, and evaluation of the radiation safety program. Radiation monitoring devices, including personal dosimeters for radiation workers, underpin the precise tracking of radiation exposure, permitting timely identification and rectification of risk factors [61,62]. Regular assessments of radiation levels within facilities further enable real-time feedback and responsive action.

Moreover, quality assurance (QA) programs and consistent equipment maintenance are crucial for the effective operation of medical imaging devices, reducing radiation exposure and ensuring patient safety. This requires a systematic approach, including the creation of protocols, performance indicators, and regular audits to assess compliance with standards. Implementing QA programs means standardising calibration, quality control, and routine maintenance procedures for imaging equipment to ensure they function within set parameters [49]. Regular audits assess image quality, dose optimisation, and procedural adherence, crucial for maintaining high patient care standards.

This intricate system of administrative and regulatory approaches coalesces to ensure optimal safety in the deployment of ionising radiation, allowing medical imaging to continue serving as an indispensable tool in modern healthcare. A robust commitment to these procedures maintains the equilibrium between diagnostic necessity and patient safety, upholding the utmost standard of radiological care.

Future perspectives and research directions

Novel Low-Radiation Imaging Modalities

Emergent low-radiation imaging techniques signify a transformative stride in medical imaging, delicately balancing radiation dosage reduction and image quality enhancement. Predominantly, low-dose computed tomography (LDCT) affords superior tomographic visualisation, facilitated by the advances in iterative reconstruction algorithms, albeit at a minor expense of image noise [63].

Digital tomosynthesis, interlacing conventional radiography and CT, capitalises on X-ray source movement to capture low-dose projections, fostering three-dimensional imaging data reconstruction. Its application has seen a marked increase in orthopaedics, chest imaging, and mammography, owing to improved lesion detection, characterisation, and superimposed structure impact reduction [64].

Revolutionising the field, photon-counting detectors present superior image quality, enhanced spatial and contrast resolution, and material-specific imaging capabilities, albeit demanding attention to implementation complexities [65]. Concurrently, photon counting detectors (PCDs) and the application of artificial intelligence, particularly deep learning-based denoising methods, have shown a noteworthy enhancement in image quality in CT and mammography, while substantially lowering radiation doses [65].

Model-based iterative reconstruction (MBIR) techniques, surpassing traditional methods, enable significant dose reduction without compromising image quality compared to traditional filtered back-projection methods [66]. Furthermore, dual-energy CT (DECT) permits simultaneous image acquisition at varied energy levels, facilitating tissue differentiation and potentially eliminating the necessity for additional scans [66].

Despite these advances, the ongoing quest for technological advancements, diagnostic efficacy evaluation, and clinician education remains integral to these modalities' effective implementation.

Artificial Intelligence and Machine Learning

Artificial intelligence (AI) and machine learning (ML) technologies are gaining significant traction in medical imaging, specifically for optimising radiation doses whilst preserving image quality [63]. This optimisation is critical, given that unwarranted exposure to ionising radiation potentially escalates cancer risk and other deleterious health outcomes. In this milieu, AI algorithms and ML models are utilised to enhance image quality, personalise imaging protocols, and forecast radiation-induced effects.

AI's noteworthy contribution to dose optimisation involves deploying deep learning algorithms for image reconstruction and noise reduction. This practice facilitates lower radiation doses without sacrificing image quality [67]. Convolutional neural networks (CNNs) have shown effectiveness, especially in recognising intricate patterns of noise and artefacts from a vast image dataset [68]. When trained on a diverse range of clinical cases, AI algorithms extend their denoising competencies to novel situations, substantially improving image quality at reduced doses.

The AI-driven personalisation of imaging protocols is another crucial aspect of dose optimisation. This ensures patients receive the lowest feasible dose while still procuring diagnostically pertinent information [69]. ML models can scrutinise patient-specific factors such as age, weight, and disease characteristics to determine the optimal scanning parameters and consequently curtail the radiation dose [70]. Furthermore, AI can assist in choosing appropriate imaging modalities, further minimising patient exposure to ionising radiation [70].

Predicting radiation-induced effects is an additional promising avenue for AI and ML. By leveraging large-scale clinical datasets, ML models can discern patterns and correlations between radiation dose and various adverse outcomes, such as cancer risk or radiation-induced tissue damage. These predictive models can be integrated into clinical decision support systems, guiding physicians in weighing the diagnostic benefits against the potential risks of radiation exposure [71].

Long-Term Risk Assessment in Large Patient Cohorts

Long-term follow-up investigations in expansive patient cohorts remain critical for elucidating the health ramifications of low-dose radiation exposure resultant from medical imaging procedures. These investigations provide critical insights into protracted risks subsequent to varied imaging modalities, thus informing clinical decision-making and radiation protection approaches. The increasing intrigue in novel technologies like iterative reconstruction algorithms and photon-counting, energy-resolved CT necessitates persistent epidemiological research [72].

A prominent feature of cohort studies is the capacity to evaluate the cumulative radiation dose from repeated imaging procedures - a scenario common in patients with chronic ailments necessitating frequent imaging. In this vein, broad cohort studies have revealed a dose-response association between cumulative radiation exposure and cancer risk, even at low doses [73]. These studies have highlighted the necessity to consider age, sex, and other factors, as they may modulate the biological response to radiation [74].

Given the inherent uncertainties in estimating long-term risks from low-dose radiation, methodological advancements in broad cohort studies are crucial. Noteworthy innovations encompass the use of advanced statistical techniques, such as Bayesian hierarchical models and ML algorithms, capable of augmenting the precision and validity of risk estimates [75]. The integration of radiogenomics and molecular epidemiology methodologies can further deepen our understanding of the biological mechanisms underpinning radiation-induced health impacts, facilitating the evolution of personalised risk assessment models [76].

The continual evolution of medical imaging technologies, together with the advent of sophisticated analytical techniques, underscores the importance of ongoing epidemiological studies. As the field advances, the findings will not only enrich our understanding of radiation risks but also guide future developments in radiation protection and clinical practice.

## Conclusions

This comprehensive review unearths salient points central to the discourse around radiology's indispensable role and its potential risks. In the exploration of ionising radiation's diverse applications, it highlights the pivotal balance between diagnostic necessity and patient safety, underscoring the 'Radiation Paradox'. The various imaging modalities, from traditional X-rays to cutting-edge nuclear medicine, demonstrate diagnostic radiation's ubiquity and inherent complexity. Simultaneously, the inherent health risks related to radiation exposure, both deterministic and stochastic, are meticulously appraised.

The findings illuminate the compelling necessity for proactive risk assessment and effective minimisation strategies in medical imaging. This is echoed by the scrutiny of radiation protection strategies and the pivotal role of the ALARA principle, presenting a multifaceted approach encompassing justification, optimisation, reference levels, protection measures, and crucially, robust regulatory guidelines.

The contributions underscore the urgency of an ongoing dialogue around radiation safety, advocating for continual reassessment and refinement of radiological practices. This review elucidates the complex interplay of benefits and risks inherent in radiology, contributing significantly to the understanding of radiation safety. Ensuring a safer future in medical imaging necessitates an unceasing dedication to this vital conversation, reaffirming the paramountcy of patient safety.
